# Response to: Comment on “Controversies about Interspinous Process Devices in the Treatment of Degenerative Lumbar Spine Diseases: Past, Present, and Future”

**DOI:** 10.1155/2017/1504316

**Published:** 2017-09-18

**Authors:** Roberto Gazzeri, Marcelo Galarza, Alex Alfieri

**Affiliations:** ^1^Department of Neurosurgery, San Giovanni-Addolorata Hospital, Rome, Italy; ^2^Regional Service of Neurosurgery, “Virgen de la Arrixaca” University Hospital, Murcia, Spain; ^3^Klinik für Neurochirurgie und Wirbelsäulenchirurgie, Neuruppin, Germany

We appreciate the comments of Dr. Landi et al. on our recent article about controversies concerning interspinous process devices (IPDs) in the treatment of degenerative lumbar spine diseases [1, 2]. We agree with a few comments of the authors: the purpose of our paper is to provide a comprehensive overview about the different interspinous implants, evaluating their mechanisms of action, their safety and cost-effectiveness in the treatment of lumbar stenosis and degenerative disc disease, and reviewing all the international published articles on this topic.

We know that, with the absolute growth of the aged population in industrialized countries, the prevalence of spinal lumbar diseases and chronic back pain is also growing and so the number of elderly patients requiring spine surgery continues to increase [3, 4]. The most frequent spinal condition in the aged population is lumbar spinal stenosis (LSS), which generally becomes symptomatic after the age of 50.

The first author to mention neurogenic claudication secondary to lumbar stenosis was Verbiest: this neurological syndrome is usually manifested by bilateral radicular pain that is exacerbated by standing, walking, and other positions that place the lumbar spine in extension. A flexed position of the lumbar spine improves or relieves the symptoms [5]. Pathologic progression of the lumbar spine begins with degenerative changes within the disc, which may lead to loss of disc height. This may result in instability that worsens the spondylosis, inducing facet joint hypertrophy. There is also hypertrophy and, more importantly, a buckling of the ligamentum flavum, mainly during extension, which contribute to the decrease of the spinal canal area and thus reducing the space available for the thecal sac and cauda equina. But there is an improved cross-sectional area with flexion [6, 7]. The progressive degeneration of a lumbar disc leads to a reduction in motion and does not lead to an increase in mobility that would be expected if the degenerative spinal process led to instability, as stated in the letter of Landi et al. The degenerated lumbar disc increases the stress experienced by the annulus, secondary to altered transmission of forces. The increased stress on the annulus is posture dependent and is one of the main causes of “mechanical back pain.” The clinical manifestations of spinal stenosis can not only limit a patient's mobility and walking ability, but also lead to depression and isolation [8]. Treatment usually starts with nonoperative approaches, such as nonsteroidal anti-inflammatory medication, oral steroids, epidural steroid injections, and physical therapy.

But conservative treatment is not effective in the long-term, and surgery is considered the only treatment option for improving quality of life and health status in the majority of cases [9]. Surgical treatment of lumbar stenosis for older patients is the most commonly performed surgical procedure in the spine, and such surgeries can be more or less invasive and a patient's age often influences the surgical decision.

There is a disagreement whether surgery in older patients entails a higher risk. Numerous articles report an age-related increase of surgical and general complications, and quoted complication rates in older patients after surgical treatment of lumbar spinal stenosis range between 2.5 and 80% [10, 11]. There is a significant association between surgery time and the occurrence of complications [12]. The initial development of interspinous devices was focused on creating a dynamic standalone treatment modality for neurogenic claudication that would not require concurrent open decompression surgery.

The surgical insertion of an interspinous device between the spinous processes is extremely simple and fast, so with its use in older patients prolonged surgery is avoided, thus avoiding major complications related to extended surgical time. Furthermore, interspinous devices can also be implanted using a mini-invasive or percutaneous approach under local anesthesia. This is one of the main reasons that has led to a boom of the use of interspinous devices in the last few years for a wide range of lumbar pathologies.

Evidence-based spine trials regarding lumbar interspinous devices are sparse. Nonspecific indications have led to controversy: a better scientific grounding is needed among spine surgeons when using these devices, and this was of the main reasons for the article that we published. The authors had no conflict of interests and we wanted to review and set the gold standard for specific indications of lumbar interspinous implants. Although indications of ISP devices include lumbar canal stenosis, degenerative spondylolisthesis (Grade I), discogenic low back pain, facet syndrome, and lumbar disc herniation, proper indications are required for interspinous devices that have such a widespread use.

Interspinous dynamic stabilization was defined as “a system that would alter favorably the movement and load transmission of a spinal motion segment, without the intention of fusion of the segment” [13]. The concept of an interspinous implant to induce flexion in the lumbar spine was introduced many decades ago, in the 1950s, with the Knowles device. Successively, Sénégas developed an interspinous implant in the 1980s, but his first report was in 1988 and then another study was in 1991 [14].

The interspinous devices currently in the spinal market are classified into two main groups: motion preservation devices and devices that fuse the interspinous space.

The motion preservation devices are further subdivided into devices that oppose the extension in a rigid manner (statics) and devices that oppose it in a flexible manner (dynamic or noncompressible) [2].

Static devices include X-Stop and Wallis implants, which are made up of noncompressible material such as metal or synthetic polymers. The static interspinous devices produce a constant amount of distraction between the spinous processes due to their noncompressible nature. Instead, dynamic devices have a major degree of compressibility. One example of a dynamic device is Coflex, formally known as interspinous “U”: it is a U-shaped piece of metal that is interposed in a compressed mode between the spinous processes and it exerts a distraction force between the two spinous processes.

Although these devices display very different biomechanical properties, they have the same mechanism of action: they act as a wedge between the spinous processes. ensuring a distraction force during extension. Due to their material and shape, the biomechanical characteristics of the flexible devices are very different, but they have a higher level of elasticity with respect to static devices, and they act as a “shock absorber” due to their deformation during extension of the spinal segment in which they are implanted.

In recent years, a new “family” of interspinous devices as a new potential fixation technique has been developed: the fusion devices. Fusion devices range from paired plates with teeth to U-shaped devices with wings that are attached to the spinous process. They were developed as an alternative to pedicle screws and rod constructs in conjunction with interbody fusion for spine fixation. They can be also used alone with the intent to fuse posteriorly two adjacent spinal segments; interspinous fixation systems are intended to be less invasive and present fewer risks than pedicle or facet screws. However, there is a lack of high-quality studies to clarify their long-term efficacy and the right indications for their use.

Both interspinous fusion devices and interspinous rigid and dynamic devices share similar characteristics, as structures or shapes. The main example is the Coflex-F spacer, which is an interspinous process device with rivets modified from the original Coflex device.

Interspinous fusion devices also share similar advantages with interspinous dynamic and static devices: a lower estimated blood loss, shorter operative time, and no significantly different hospital length of stay. In a meta-analysis performed by Cai et al. comparing interspinous fusion devices and Posterior Lumbar Interbody Fusion PLIF, clinical outcomes of interspinous spacers were equivalent to or even superior to PLIF, with a minor complication rate [15].

*Mechanism of Action*. Biomechanically, interspinous devices act to limit extension and have no effect on flexion, axial rotation, or lateral bending; they reduce the degree of thecal sac impingement due to buckling of the ligamentum flavum, stretching it with tension, and enlarging the spinal canal area. Moreover, interspinous devices act to offload the facet joints, acting like a “shock absorber,” and dissipate forces dorsally [16]. Furthermore, IPDs reduce intervertebral disc pressures, particularly in the region of the posterior endplate; Swanson examined the changes in intradiscal pressures at the level of the implant during flexion and extension: when the lumbar spines were in a neutral or extended position, there was a significant decrease in the intradiscal pressure and the pressure at the posterior annulus of the implanted segment [17].

 Richards et al. studied the effects of the X-Stop interspinous spacer on the dimensions of the areas of the spinal canal and neural foramina: in extension, the implant increased the canal area by 18%, the subarticular diameter by 50%, and the canal diameter by 10%; moreover, the foraminal area and the foraminal width were increased by 25% and 41%, respectively [17]. Another study using the X-Stop device reported that the cross-sectional foraminal area at the implanted level was increased by 36.5% and the spinal canal had a mean expansion of 22% after insertion of interspinous device [18]. Siddiqui et al. also observed that the X-Stop device implantation enlarged the foraminal area in extension, with a 20% increase [18]. In another study in 2012, the authors reported a significative increase of foraminal cross-sectional area (from 125.91 preoperatively to 148.17 mm2 at last followup assessment) using the Aperius device: the mean increase was 17.60% of average foraminal area [19].

Landi et al. suggest that a posterior distraction of the spinous processes secondary to IPD insertion forces the dislocation of the nucleus pulposus posteriorly, theoretically increasing the risk of disc herniation. For this reason, the tension bands used in several IPDs such as Diam, Viking, and Wallis permit a direction of the distraction that is parallel to the vertebral endplates, so that the intradiscal pressure can be reduced in a homogeneous fashion. The tension band associated with the interspinous implant generates a posterior and anterior unload of the disc. Biomechanical studies on an interspinous fusion device (Aspen) confirmed that Interspinous Fusion Device (IFD) can provide an increase in foraminal height, with a similar mechanism to treating neurogenic symptoms with a dynamic device [20, 21].

We suggest that the use of a big device is associated with a great increase of the dimension of the spinal canal and foramen and usually it should be used in the treatment of patients with lumbar spinal stenosis. But, overdistraction of the interspinous process may induce an excessive load on the anterior annulus, thus accelerating disc degeneration. The choice of the correct implant size is extremely important for the patient's clinical outcome. To achieve correct implantation and to avoid an overestimation of the device size, we recommend always using the device templates and preoperatively measuring the distance between the spinous processes.

*Interspinous versus Laminectomy*. We reviewed many studies comparing interspinous device implantation versus laminectomy for the treatment of lumbar spinal stenosis. Stucki et al. reported outcomes in a series of almost 200 patients treated with a lumbar laminectomy versus X-Stop, demonstrating higher clinical success rates in interspinous devices compared with the laminectomy patients [21]. Turner et al. reported an evaluation of the cost and effectiveness of X-Stop and laminectomy surgery [22]. X-Stop was demonstrated to be significantly more cost-effective compared with laminectomy for the treatment of single- and two-level LSS and there were no surgical complications in the X-Stop group, with a shorter operative time when compared with the open decompression group. Data from the literature suggest that interspinous device surgery may be preferred in older, unstable patients with comorbidities.

*Complications*. We are aware of several reports about complications and failure rates of interspinous devices, and one has been recently published by us, Gazzeri et al. [23]. In this European multicenter study, 1108 consecutive patients between January 2002 and January 2012 underwent IPD placement. The overall reoperation rate was 9.6%: 63 cases required instrumented fusion with pedicle screws, 24 the removal of the implant and decompressive surgery, 12 insertion of a bigger interspinous spacer, and 8 the removal of the interspinous implant; in 13 cases removal of the implant secondary to failure was within the first 3 months after surgery, while in 94 cases implants were removed after a minimum of 2 years.

 Verhoof et al. reported a high failure rate of X-Stop interspinous distraction, defined as surgical reintervention, in patients with lumbar stenosis caused by spondylolisthesis: redo surgery was required in 58% of patients within 2 years [24].

In another study, patients with spinal stenosis secondary to lumbar spondylolisthesis, treated with X-Stop, were compared to patients with nonoperative treatment; the overall clinical success rate was 63.4% was in the X-Stop group and only in 12.9% in the conservative group, but at two-year follow-up surgical reintervention was required in 11.9% of the patients in the IPD group compared to 12.1% in the conservative group [25]. In another study, 80% of patients with Grade I spondylolisthesis required additional surgery after IPD placement.

For this reason we and other authors support the recommendation that interspinous devices should not be used in patients with spondylolisthesis [26].

In another recent paper, we evaluated a large series of 422 patients who underwent surgical treatment consisting of X-Stop device implantation; in this series twenty-five intraoperative (4.9%) and forty-seven postoperative (11.1%) complications were noted, but no neurologic adverse events were reported [26]. There were 16 intraoperative spinous process fractures probably caused by the wrong position of the distractor instrument used to distract the spinal processes and to determine the adequate size of the implant. Furthermore, 14 of these patients had a history of osteoporosis, and the fracture was likely due to an osteopenic bone.

There were 9 craniospinal fluid leakages secondary to the low insertion of the dilator instrument inside the interspinous ligament with dural damage, and most commonly the L5-S1 level was affected. Eighteen patients experienced postoperative device dislocations correlated with pain; in all of these cases the X-Stop was removed, and a laminectomy with spinal fusion was performed; in two cases, another IPD was inserted.

*Conclusion*. As reported in several radiological and cadaveric studies, IPDs have an effect on soft central and foraminal stenosis, because their action is effective on ligamentous buckling and hypertrophy, with good clinical results on neurogenic claudication in the early stage of the postoperative period. Evidence-based spine trials regarding lumbar IPDs are sparse and nonspecific indications with overstated rejections have led to controversy, as stated by Landi et al. Indeed, a better scientific grounding is needed among spine surgeons when using these devices. The purpose of our study was to review the different interspinous devices on the market, their mechanisms of action, and their surgical indications.
